# Neuromodulatory Interventions in Experimental Acute Pancreatitis: A Systematic Review of Rodent Studies

**DOI:** 10.3390/diseases14040145

**Published:** 2026-04-16

**Authors:** Maxim Rantsev, Alexey Sarapultsev, Valeriy Chereshnev

**Affiliations:** 1 Institute of Immunology and Physiology, Ural Branch of the Russian Academy of Science, 106 Pervo-Maiskaya Street, 620049 Ekaterinburg, Russia; r-ma@bk.ru (M.R.);; 2 Department of Surgical Diseases, Ural State Medical University, 620028 Ekaterinburg, Russia; 3 Russian–Chinese Education and Research Center of System Pathology, South Ural State University, 454080 Chelyabinsk, Russia

**Keywords:** acute pancreatitis, serotonin, 5-HT2A receptor antagonism, neuroimmune modulation, hypothalamic–pituitary–adrenal axis, ferroptosis, rodent models, systematic review

## Abstract

**Background/Objectives**: Acute pancreatitis (AP) lacks disease-modifying pharmacotherapy. Neuroimmune, serotonergic, and redox-regulated pathways may modulate inflammatory amplification and acinar injury, although pharmacovigilance data link some psychotropic drug classes to AP risk. This review synthesized controlled rodent studies evaluating neuromodulatory interventions with serotonergic, stress-axis, or ferroptosis-linked targets in experimental AP. **Methods**: PubMed, Scopus, eLIBRARY.ru, and Elicit were searched in January 2026, supplemented by Google Scholar audit and citation chasing. Eligible studies were controlled in vivo rodent experiments using validated AP models with quantitative outcomes. Intervention timing was classified a priori as a primary analytic variable. Risk of bias was assessed with SYRCLE. A prespecified audit showed that no subset met the criteria for quantitative pooling because of heterogeneity in model class, compounds, timing, outcome definitions, units, and sampling timepoints. Mechanism-stratified qualitative synthesis was therefore performed. The protocol was registered on OSF (doi: 10.17605/OSF.IO/CZXDJ). **Results**: Nine studies (1992–2023) yielded 410 outcome rows across three mechanistic strands. Serotonergic modulation (5-HT_2_/5-HT_2_A-focused; six studies) reduced serum amylase/lipase (−37% to −65% vs. disease controls) and histological injury, with receptor-selectivity data supporting 5-HT_2_A-mediated mechanisms. Stress-axis modulation with thiadiazine L-17 reduced 7-day mortality in two severe models (from 50–70% to 30%). Olanzapine attenuated ferroptosis-linked injury via off-target antioxidant activity independent of serotonergic receptors. All interventions were prophylactic, peri-induction, or very early post-induction; no delayed therapeutic-window studies were identified. Most SYRCLE domains were unclear, particularly allocation concealment and blinding-related procedures. **Conclusions**: Neuromodulatory pathways modulate experimental AP in rodents, but evidentiary strength differs across mechanistic strands. Inference is constrained by absent therapeutic-window testing, heterogeneous endpoints, and reporting deficits. The findings support mechanism-level target prioritization rather than clinical repurposing.

## 1. Introduction

Acute pancreatitis (AP) is an acute inflammatory disorder of the exocrine pancreas that can evolve from a self-limited local injury to a systemic inflammatory response syndrome with organ dysfunction. Contemporary clinical definitions and severity strata are anchored in the Revised Atlanta Classification, in which persistent organ failure (>48 h) is the defining criterion of severe disease and is strongly associated with mortality [1]. Consequently, major clinical guidelines treat AP as a time-critical condition in which early risk stratification and prompt supportive care are central to outcome [1,2,3].

At the population level, AP represents a substantial emergency and inpatient burden, and epidemiology indicates rising incidence in many regions. Population-based European data show sustained increases in AP incidence over time and marked cross-country heterogeneity, consistent with differences in etiology (gallstone disease, alcohol, and metabolic risk) and case ascertainment [4]. Global burden analyses similarly report increasing pancreatitis incidence and attributable mortality over recent decades [4,5,6,7]. In the United States, pancreatitis contributes materially to direct medical expenditures among digestive diseases, underscoring the public-health relevance of interventions capable of reducing systemic complications and length of stay [5].

Despite extensive preclinical exploration of cytokine blockade, antioxidants, enzyme inhibition, and diverse pathway-targeted candidates, there remains no pharmacological agent with reproducible, guideline-endorsed disease modification across etiologies and severity strata. Accordingly, authoritative guidance emphasizes early fluid resuscitation, analgesia, early enteral nutrition when feasible, judicious use of imaging, and etiology-directed interventions (e.g., biliary decompression when indicated), rather than targeted anti-inflammatory pharmacotherapy [2,3]. This persistent therapeutic gap motivates reassessment of mechanistically plausible, clinically tractable approaches that could attenuate inflammatory amplification rather than the initiating acinar insult. The immunopathogenesis of AP—encompassing acinar cell injury, immune cell activation, cytokine cascades, and progressive tissue destruction—has been extensively characterized at the cellular and molecular level [8], providing a rich landscape of potential intervention targets.

Neural regulation of peripheral immunity is now an established biological principle. The “inflammatory reflex” and the cholinergic anti-inflammatory pathway describe afferent inflammatory sensing and efferent autonomic outputs that suppress cytokine production via acetylcholine-dependent signaling, including α7 nicotinic acetylcholine receptor-mediated inhibition of macrophage pro-inflammatory responses [9,10,11]. This framework is not purely conceptual: recent clinical translation of bioelectronic neuromodulation for inflammatory disease illustrates that targeted manipulation of vagal circuits can yield measurable anti-inflammatory effects in humans [12].

For AP, the neuroimmune framework is conceptually compelling because disease severity is shaped not only by initiating acinar cell injury but also by secondary systemic responses, including cytokine cascades, endothelial activation, leukocyte trafficking, microcirculatory dysfunction, and organ cross-talk. A coherent translational hypothesis therefore follows: interventions with central–peripheral neuromodulatory activity may attenuate AP severity by damping systemic inflammatory amplification and stabilizing microvascular and barrier functions during the critical early phase when organ failure trajectories are established.

Serotonin (5-hydroxytryptamine; 5-HT) is not only a central neurotransmitter; most whole-body 5-HT is synthesized peripherally (predominantly by enterochromaffin cells) and stored in platelets, with broad effects on vascular tone, endothelial function, immune cell trafficking, and cytokine signaling [13,14]. These actions are receptor-specific and context-dependent, and 5-HT receptors are expressed across multiple immune cell classes, enabling direct modulation of inflammatory responses [14]. In inflammatory settings, 5-HT_2_ family signaling has been repeatedly implicated in pro-inflammatory and pro-thrombotic vascular phenotypes, providing a mechanistic bridge to agents with 5-HT_2_A antagonism [13,14]. Early tissue-level evidence further supports serotonergic involvement in the pancreatic injury milieu: Celiński et al. (1995) documented significant reductions in pancreatic 5-HT and 5-HIAA levels during caerulein-induced AP in rats, consistent with local serotonin release and turnover during the acute inflammatory phase [15].

Within experimental pancreatitis, a focused preclinical literature has repeatedly tested whether serotonergic modulation alters biochemical and histological indices of injury. Classical 5-HT_2_ receptor antagonists (e.g., ketanserin) were evaluated in hyperstimulation-based AP models, and subsequent studies assessed selective 5-HT_2_A antagonists and clinically used drugs with prominent 5-HT_2_A antagonism (e.g., risperidone) in edematous and severe necrotizing paradigms [16,17,18,19,20]. More recent work has also leveraged modern screening concepts (e.g., ferroptosis modulation) to implicate centrally active drugs in pancreatic protection, suggesting that neuromodulatory pharmacology may intersect with tissue-injury programs beyond canonical neurotransmission [21].

Neuromodulatory drugs are heterogeneous, but many have well-characterized profiles at serotonergic and related targets with known immunovascular consequences. A systematic synthesis focused on in vivo rodent AP models can therefore serve two functions: first, to consolidate a dispersed literature into a coherent mechanistic map (target → pathway → organ outcome); and second, to clarify whether reported effects are consistent across models and endpoints or restricted to specific paradigms, timings, and dosing schedules.

A translational tension must be acknowledged a priori. Pharmacoepidemiologic data on psychotropic drugs and acute pancreatitis are heterogeneous and appear to depend on drug class, individual agents, and study design. Population-based case–control and spontaneous-report analyses have described signals of increased risk for antidepressants overall and for selected classes or agents, although confounding by indication, alcohol use, metabolic comorbidity, and polypharmacy is difficult to fully exclude [22,23,24,25,26]. A recent meta-analysis of case–control studies found a modest association between SSRI use and AP risk [27], while a comprehensive systematic review and meta-analysis of psychotropic drugs broadly confirmed heterogeneous risk signals across drug classes [28]. By contrast, a large Danish register-based cohort specifically comparing fluoxetine with citalopram and other SSRIs did not detect an increased risk among fluoxetine users [24]. Taken together, these studies argue against a uniform, class-wide “toxic” effect and instead support a more nuanced interpretation in which modest drug-specific effects, background risk, and residual confounding jointly shape the observed epidemiologic signals [22,23,24,25,26].

The aim of this review was to identify, stratify, and critically synthesize in vivo rodent studies of experimentally induced acute pancreatitis in which neuromodulatory interventions with defined serotonergic, stress-axis, or ferroptosis-linked pharmacology were administered, and to evaluate whether reported effects remained coherent after explicit consideration of mechanism, intervention timing, and endpoint structure.

The central research question is: in rodent models of experimentally induced acute pancreatitis, do neuromodulatory agents with defined serotonergic/stress-axis/ferroptosis-linked activity alter pancreatic injury severity and systemic inflammatory outcomes compared with pancreatitis controls, and are reported effects consistent with mechanistically distinct pathways?

## 2. Materials and Methods

### 2.1. Protocol, Reporting Framework, and Review Design

This review was designed as a structured systematic synthesis of controlled in vivo rodent studies evaluating neuromodulatory interventions with defined serotonergic, stress-axis, or ferroptosis-linked targets in experimentally induced acute pancreatitis. The methodological structure, screening logic, and reporting were aligned with PRISMA 2020 principles for transparent study identification and selection. Risk of bias was assessed using SYRCLE’s risk-of-bias tool for animal studies with three-level judgments (Low/Unclear/High). Given the anticipated sparsity and heterogeneity of quantitative data across models, interventions, timing schedules, and outcome panels, a mechanism-stratified qualitative synthesis was planned a priori as the default analytic framework, with quantitative pooling considered only when outcome definitions, timing, and reporting permitted defensible aggregation. Quantitative meta-analysis was not performed (see Section 2.8 for eligibility criteria and rationale). The review protocol was registered a priori on the Open Science Framework (OSF) (doi: 10.17605/OSF.IO/CZXDJ). No major post hoc changes to the review question or eligibility criteria were introduced after registration.

Terminology note: To avoid conflating pharmacodynamically distinct intervention classes under a single label, we use “neuromodulatory interventions” as the neutral analytic umbrella term and reserve “psychotropic” for contextual pharmacovigilance discussion. Three mechanistic strata are treated as distinct throughout: (i) 5-HT_2_/5-HT_2_A receptor-mediated serotonergic antagonism; (ii) stress-axis/neuroimmune modulation (including the substituted thiadiazine compound L-17); and (iii) ferroptosis-linked modulation (olanzapine, treated as a pleiotropic/off-target proof-of-principle rather than a serotonergic receptor effect).

### 2.2. Eligibility Criteria

Eligible records were controlled in vivo studies in rodents (rats or mice) using validated experimental models of acute pancreatitis or necrotizing/severe acute pancreatitis (e.g., cerulein/caerulein hyperstimulation, duct ligation, choline-deficient ethionine-supplemented diet, post-manipulation models). Interventions of interest were neuromodulatory agents with defined serotonergic, stress-axis, or ferroptosis-linked pharmacology (including anti-psychotics/neuroleptics, antidepressants, selective serotonergic receptor antagonists/agonists, serotonin transporter-oriented modulators, and agents identified through ferroptosis-focused screening) administered with the intent to modify pancreatitis severity or systemic inflammatory consequences. Required outcomes included at least one quantitative pancreatitis endpoint: serum amylase and/or lipase; pancreatic histology/necrosis scoring; inflammatory cytokines; mortality; or defined systemic organ injury endpoints. Exclusions were (i) purely in vitro work, (ii) human case reports/clinical series, (iii) drug-induced pancreatitis as an adverse event without an experimental pancreatitis model, (iv) LPS-driven systemic inflammation models used as a surrogate for pancreatitis, and (v) studies without analyzable quantitative endpoints.

### 2.3. Information Sources and Search Dates

Systematic searches were conducted on 4 January 2026 in PubMed and Scopus, complemented by targeted retrieval from eLIBRARY.ru and a supplementary semantic search via Elicit. To assess coverage beyond these databases, a targeted Google Scholar audit was performed using PICO-constrained queries and systematic forward/backward citation chasing from all nine included studies and key review-level sources. Full-text retrieval and data extraction were updated through 30 January 2026 as additional PDFs became available.

### 2.4. Search Strategy

The core search logic combined disease terms (acute pancreatitis/necrotizing pancreatitis/pancreatonecrosis), rodent terms, and intervention terms (neuromodulatory drug classes and predefined drug names), optionally constrained by common experimental model keywords. The final canonical strings used for retrieval are recorded below.

PubMed (Title/Abstract + MeSH where applicable):

((“pancreatitis, acute”[MeSH Terms] OR “acute pancreatitis”[Title/Abstract] OR “necrotizing pancreatitis”[Title/Abstract] OR pancreatonecrosis[Title/Abstract] OR pancreatitis[Title/Abstract]) AND (rat[Title/Abstract] OR rats[Title/Abstract] OR mouse[Title/Abstract] OR mice[Title/Abstract] OR rodent[Title/Abstract]) AND (antipsychotic[Title/Abstract] OR neuroleptic[Title/Abstract] OR antidepressant[Title/Abstract] OR serotonergic[Title/Abstract] OR “5-HT2”[Title/Abstract] OR ketanserin[Title/Abstract] OR spiperone[Title/Abstract] OR risperidone[Title/Abstract] OR metergoline[Title/Abstract] OR ritanserin[Title/Abstract] OR methysergide[Title/Abstract] OR olanzapine[Title/Abstract] OR tropisetron[Title/Abstract])) NOT (case reports[Publication Type] OR review[Publication Type])

Scopus (TITLE-ABS-KEY):

TITLE-ABS-KEY((“acute pancreatitis” OR “necrotizing pancreatitis” OR pancreatitis OR pancreatonecrosis) AND (rat OR rats OR mouse OR mice OR rodent) AND (antipsychotic OR neuroleptic* OR antidepressant* OR serotonergic OR “5-HT2” OR ketanserin OR spiperone OR risperidone OR metergoline OR ritanserin OR methysergide OR olanzapine OR tropisetron)) AND (LIMIT-TO(DOCTYPE, “ar”))

eLIBRARY.ru: Russian-language equivalent queries.

Elicit: Supplementary semantic search constrained to validated AP models and neuromodulatory/psychotropic drugs; screened under the same eligibility criteria.

Coverage audit: Targeted Google Scholar queries and forward/backward citation chasing from all included studies (n = 9) and key backbone sources. The audit did not identify additional eligible intervention studies; one context-only biomarker anchor (Celiński et al. 1995) was identified and is cited as background evidence for serotonergic involvement but was not included in the intervention corpus [15].

### 2.5. Selection Process and PRISMA Accounting

**Figure 1:**
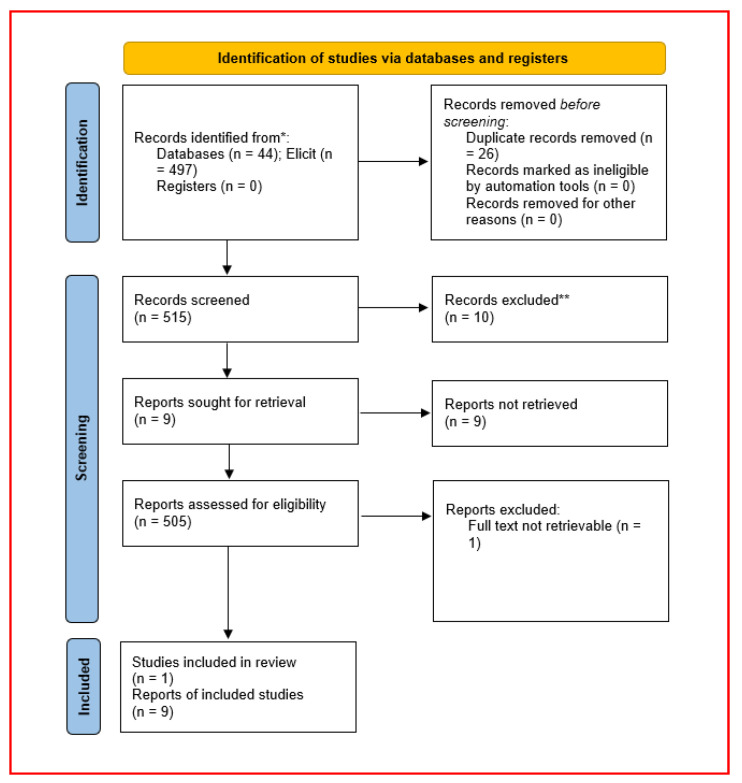
PRISMA 2020 flow diagram for study identification and selection. Records identified: n = 541 (bibliographic databases n = 44; Elicit semantic search n = 497). Duplicates removed: n = 26. Records screened: n = 515. Records excluded at title/abstract: n = 505 (primarily non-neuromodulator exposures, non-pancreatitis models, human/case-report designs, or absence of quantitative endpoints). Full texts sought: n = 10. Full text not retrieved: n = 1 (Chinese-language report; retained as contextual background [29]). Reports assessed for eligibility: n = 9. Studies included in qualitative synthesis: n = 9. * Records identified from Elicit represent results of a supplementary semantic search used for retrieval support; inclusion and exclusion decisions were based on manual screening according to predefined eligibility criteria. ** Records excluded at title/abstract screening include studies with non-neuromodulatory exposures, non-pancreatitis models, human or case-report designs, or absence of quantitative outcomes.

Across all sources, 541 records were identified (44 from bibliographic databases and 497 from the Elicit export). After deduplication (26 removed by DOI and bibliographic matching), 515 records underwent title/abstract screening; 505 were excluded primarily for non-psychotropic exposures, non-pancreatitis models, human/case-report designs, or absence of quantitative endpoints. Ten full texts were sought; nine full-text studies were assessed and included in qualitative synthesis. One Chinese-language experimental report evaluating chlorpromazine in a rat model of acute necrotizing pancreatitis (Li Qiang et al., 2008) was identified as potentially eligible, but the full text could not be obtained and was retained as contextual background only [29]. Two reviewers independently screened all records at the title/abstract stage and independently assessed retrieved full texts for eligibility. Discrepancies were resolved by discussion and consensus. Elicit was used only for retrieval support and not for inclusion/exclusion decisions. The selection pathway is summarized in Figure 1.

### 2.6. Data Extraction and Harmonization

Data were extracted into predefined structured templates capturing species/strain/sex, model induction details, intervention dosing and timing, group sizes, outcome definitions and units, summary statistics (mean with dispersion), and analytic notes. Primary extraction was performed by one reviewer and independently checked by a second reviewer; discrepancies were resolved by discussion and consensus. Authors of primary studies were not contacted for missing data.

Timing stratification. As a primary analytic variable, each intervention arm was classified into one of four timing categories: (i) pre-induction (administration completed before pancreatitis induction); (ii) peri-induction (administration overlapping with or beginning simultaneously with induction); (iii) early post-induction (first dose within hours of induction onset); (iv) delayed post-induction therapeutic (first dose ≥ 24 h after established disease). Timing category and details are reported for each intervention arm in Table 1.

Figure digitization. When numerical outcomes were reported only graphically and were central to the synthesis, figure digitization was performed using high-resolution PDF renders. All digitized values are flagged with provenance metadata (figure/table locator, digitization method) in the extraction tables. A digitization audit (available from the authors) reports coverage by study. Digitization cannot replace full numeric reporting when dispersion measures or group sizes are not recoverable from figures, and this constraint is noted where relevant.

Standardized effect computation. For each endpoint with a computable disease-control comparator (matching study × model × endpoint × timepoint × matrix × unit), percent change relative to disease control (%Δ) was calculated as %Δ = (Mean_intervention − Mean_disease_control)/Mean_disease_control × 100. Non-computable cases are left blank.

### 2.7. Risk of Bias Assessment

Risk of bias was evaluated using SYRCLE’s tool across standard domains (sequence generation, baseline characteristics, allocation concealment, random housing, blinding of caregivers and investigators, blinding of outcome assessors, incomplete outcome data, selective outcome reporting, and other biases). Each domain was coded as Low/Unclear/High based strictly on reporting and design features. Assessment was performed independently by two reviewers at the study level; domain-level disagreements were resolved by discussion and consensus. No automation tools were used.

### 2.8. Synthesis Strategy and Rationale for Qualitative Synthesis

Given the small number of eligible studies (n = 9), structural heterogeneity of models and outcomes, and variability in reporting completeness, we planned a mechanism-stratified qualitative synthesis as the primary analytic framework. Studies were organized by the primary target axis engaged by the intervention: (i) 5-HT_2_/5-HT_2_A receptor antagonism; (ii) stress-axis/neuroimmune modulation; (iii) ferroptosis-linked modulation. For each study, directionality of effects was summarized for predefined outcome domains (pancreatic injury indices, enzyme release, systemic inflammation, and survival/mortality).

Meta-analysis eligibility criteria. Exploratory quantitative pooling was considered for any endpoint subset meeting all of the following: (a) ≥3 independent studies contributing data on a homogeneous outcome (same endpoint, comparable model class, comparable timing); (b) extractable mean, variance, and group size for each study; (c) sufficient model/intervention homogeneity to permit defensible interpretation of a pooled estimate. After digitization and harmonization, a prespecified eligibility audit was conducted. No subset met all three criteria: the cerulein + serum amylase + serotonergic antagonist subset comprised three studies but involved different compounds, dose ranges, species, timepoints, and units (SU/dL vs. IU/L vs. IU/mL), precluding meaningful standardized pooling. We therefore report structured quantitative data (including %Δ vs. disease control) in outcome tables but do not present pooled effect estimates.

Publication and reporting bias. Formal small-study statistical tests (e.g., funnel plots, Egger’s test) are not meaningful at this corpus size (k = 9 overall; k ≤ 3 per mechanistic subgroup). However, qualitative assessment of publication bias risk is warranted. Animal intervention studies are known to be susceptible to the “file drawer” problem, whereby neutral or negative findings are less likely to be published. In the present corpus, all included studies reported statistically significant effects favouring the intervention on at least one primary endpoint, which is compatible with positive-result publication bias. Additionally, the absence of study preregistration or published protocols for any of the nine studies, combined with heterogeneous outcome panels, raises the possibility of selective outcome reporting. Figure-only presentation of key endpoints, observed in three studies, may further amplify reporting bias by limiting independent verification of effect magnitudes. Taken together, these considerations support a conservative interpretation: directional consistency is notable, but effect magnitudes may be inflated and the strength of evidence should not be overstated.

Certainty assessment. A formal certainty-of-evidence framework (e.g., GRADE) was not applied. Confidence in directional findings was discussed narratively with consideration of risk of bias, consistency across models, heterogeneity, and completeness of numerical reporting.

## 3. Synthesis of Included Evidence

### 3.1. Study Selection

Across all sources, 541 records were identified (bibliographic databases n = 44; Elicit export n = 497). After removal of 26 duplicates, 515 unique records underwent title/abstract screening, and 505 were excluded because they did not involve neuromodulatory/serotonergic interventions, did not use an experimental pancreatitis model, were human/case-report designs, or lacked quantitative outcomes. Ten full texts were sought for retrieval; nine full-text reports were assessed for eligibility and included in the qualitative synthesis. One potentially relevant Chinese-language experimental report evaluating chlorpromazine in a rat model of acute necrotizing pancreatitis could not be obtained in full text and was therefore retained as contextual background only [29]. The targeted Google Scholar coverage audit and forward/backward citation chasing from all included studies did not identify additional eligible intervention studies. The selection pathway is summarized in Figure 1, and the core study characteristics are consolidated in Table 1.

### 3.2. Structure of the Evidence Base, Timing Distribution, and Extractability

The nine included studies (1992–2023) span four experimental model classes: cerulein/caerulein hyperstimulation (4 studies), choline-deficient ethionine-supplemented (CDE) diet-induced severe necrotizing pancreatitis (3 studies, two of which also used cerulein), duct ligation/proserin-based severe AP (1 study), and post-manipulation pancreatitis (1 study). One additional study used a ferroptosis-sensitized genetic background (pancreas-specific Gpx4 knockout) with cerulein induction [21].

Timing distribution. Across all intervention arms, dosing was classified as pre-induction in 5 studies ([16,17,18,19] cerulein and ligation arms), peri-induction in 3 studies ([18,20,30], CDE arm), and early post-induction in 3 studies [21,31,32]. No study evaluated delayed post-induction therapeutic dosing (first dose ≥ 24 h after established disease). This predominance of prophylactic/peri-insult timing is a structural property of the field that constrains translational inference.

Extractability. Full numerical extraction (mean ± dispersion with group sizes) was feasible for six studies from tables and text. Three studies [18,21,30] reported key outcomes primarily in figures; these were digitized with provenance. The complete extraction yielded 410 outcome rows across all studies. The %Δ vs. disease control was computable for 157 of 206 intervention rows.

Meta-analysis eligibility. Following the prespecified eligibility audit, no endpoint subset met the pooling criteria (≥3 independent studies with extractable variance on a homogeneous outcome). The cerulein + serum amylase + serotonergic antagonist candidate subset comprised three studies [16,18,30] but these involved different compounds (ketanserin, tropisetron, R-102444), species (rat vs. mouse), units (SU/dL, IU/L, IU/mL), and timepoints, precluding defensible standardized pooling.

Pooling criteria: ≥3 independent studies with extractable mean, variance, and group size on a homogeneous outcome (same endpoint, comparable model class, comparable timing). No subset met all three criteria.

A full endpoint-level extraction table with matched disease-control means and percent change values is provided as Supplementary Table S1, whereas Table 2 presents a condensed study-level quantitative profile to facilitate cross-study reading without obscuring model-specific heterogeneity.

Standardization of intervention effects relative to matched disease controls (Table 2) enables comparison of effect direction and approximate magnitude across heterogeneous models without implying cross-study equivalence. In this normalized representation, the evidence base exhibits a clear asymmetry across mechanistic strands.

Serotonergic interventions show the most consistent pattern, with reproducible reductions in enzyme-defined pancreatic injury and, in severe models, parallel improvements in histological damage and selected systemic endpoints [16,17,18,19,20,30]. This pattern is internally coherent with receptor-selectivity data and is further supported by independent evidence of tissue-specific alterations in serotonin turnover during experimental acute pancreatitis [15].

In contrast, the L-17 studies demonstrate a distinct effect profile, with the strongest signal observed in survival outcomes and shifts in inflammatory-cell composition, rather than in uniform suppression of cytokine panels [31,32]. The olanzapine study shows broad attenuation of injury markers within a ferroptosis-sensitized framework; however, this effect is mechanistically distinct and does not extend the receptor-mediated serotonergic pattern [21].

Despite normalization, the dataset remains structurally heterogeneous, with differences in model class, species, intervention timing, endpoint definitions, and measurement units. Accordingly, these quantitative summaries should be interpreted as structured comparisons of within-study effect magnitudes rather than as a basis for pooled inference.

A prespecified eligibility audit identified several candidate subsets for exploratory pooling, including cerulein-induced models with serum amylase under serotonergic antagonists, CDE-based enzyme outcomes, severe-model mortality under L-17, and ferroptosis-linked readouts under olanzapine. However, no subset met all pooling criteria simultaneously. Across these candidates, extractable variance, sufficient study number, and outcome homogeneity did not co-occur within the same analytic stratum. Limitations included small subset size (k = 1–2), inconsistent reporting formats (e.g., percentage inhibition or figure-derived data), differences in species and model class, and variability in timing and endpoint definitions. Accordingly, quantitative synthesis was not pursued.

Results are therefore organized by mechanistic axis: serotonergic antagonism (Section 3.3), stress-axis modulation (Section 3.4), and ferroptosis-linked modulation (Section 3.5).

### 3.3. Serotonergic Antagonism in Hyperstimulation and Diet-Induced Pancreatitis Models

#### 3.3.1. Cerulein Hyperstimulation Models: Dose–Response Enzyme Data

In the rat cerulein model (Oguchi 1992), serum amylase increased from 2951.6 ± 239.8 SU/dL (saline controls) to 23,043.3 ± 2570.6 SU/dL (cerulein + vehicle). Ketanserin reduced hyperamylasemia dose-dependently: 14,477.0 ± 2838.0 (1 mg/kg; %Δ = −37.2%), 12,076.2 ± 1105.8 (3 mg/kg; −47.6%), 11,129.0 ± 1679.7 (10 mg/kg; −51.7%), and 11,546.3 ± 1758.0 SU/dL (20 mg/kg; −49.9%). Ritanserin at 10 mg/kg reduced amylase to 13,318.9 ± 1021.3 SU/dL (−42.2%). Ketanserin also reduced cerulein-induced pancreatic weight gain (0.66 ± 0.01 vs. 0.74 ± 0.02 g/100 g BW in vehicle controls; %Δ = −10.8%) and improved histological changes qualitatively [16].

In the mouse cerulein model (Hamada 2007), a comprehensive receptor-selectivity analysis was performed. Among selective 5-HT_2_A antagonists, the amylase potency index (mean inhibition across 0.32 and 3.2 mg/kg doses) ranked as: risperidone (42.5%) > spiperone (34.8%) > ketanserin (29.1%) > AMI-193 (4.4%) > MDL 11,939 (−6.6%). Lipase potency indices followed a parallel pattern: risperidone (60.5%) > spiperone (41.1%) > ketanserin (38.7%). Non-selective 5-HT_2_A/_2_B/_2_C antagonists showed lower potency indices despite high receptor affinity—metergoline (23.2%/24.1%), ritanserin (9.8%/8.8%), and methysergide (−8.0%/−9.0%)—consistent with opposing vascular effects of 5-HT_2_B/_2_C blockade. Selective 5-HT_2_B (SB204741) and 5-HT_2_C (SB242084) antagonists had no effect on hyperenzymemia [19]. Pharmacological serotonin depletion with p-CPA reduced cerulein-induced amylase from 41,174 ± 2676 to 32,498 ± 1897 IU/L (200 mg/kg; %Δ = −21.1%) and 31,978 ± 2465 IU/L (400 mg/kg; −22.3%), with parallel lipase reductions [19]. Histologically, risperidone dose-dependently attenuated inflammatory cell infiltration (from 2.10 ± 0.14 to 1.40 ± 0.11 at 3.2 mg/kg) without affecting edema scores, whereas SB204741 and SB242084 had no effect on histological parameters [19].

Tropisetron (2 mg/kg), a 5-HT_3_ receptor antagonist with additional α7 nAChR partial agonism, reduced cerulein-induced serum amylase and lipase, hepatic enzymes (ALT, AST), pancreatic MPO activity, and pancreatic TNF-α and IL-1β content in mice, with histological improvement (Rahimian 2017). All endpoints showed attenuation relative to cerulein controls, with the most pronounced effects on inflammatory cytokines [30].

In the multi-model study (Ogawa 2005), the selective 5-HT_2_A antagonists R-102444 and its active metabolite R-96544 reduced serum amylase and lipase in rat cerulein, rat duct ligation, and mouse CDE models. In the CDE model, R-96544 dose-dependently attenuated not only necrosis and inflammation but also cytoplasmic vacuolization—a feature not previously reported for 5-HT_2_A antagonists [18].

#### 3.3.2. Severe Diet-Induced (CDE) Models: Enzymes, Histology, and Survival

In the CDE diet model [17], serum amylase rose from 983 ± 52 IU/L (controls) to 40,725 ± 6514 IU/L (pancreatitis, Day 3), with lipase showing a parallel pattern (45 ± 2 to 4922 ± 693 IU/L). Plasma 5-HIAA levels, reflecting serotonin release, were significantly elevated on Days 1–3 in CDE mice, preceding the enzyme and histological changes [17]. In the ketanserin dose–response arm (Day 3 survivors), amylase was 46,194 ± 8737 (vehicle), 43,750 ± 7285 (1.0 mg/kg; %Δ = −5.3%), 47,227 ± 6182 (3.2 mg/kg; +2.2%), and 18,844 ± 3560 IU/L (10 mg/kg; −59.2%). Cyproheptadine showed a different pattern: amylase was 25,600 ± 4948 (vehicle), 28,805 ± 4007 (0.32 mg/kg), 36,530 ± 8069 (1.0 mg/kg), and 20,516 ± 5967 IU/L (3.2 mg/kg; −19.9%), without reaching statistical significance. Importantly, both agents markedly attenuated histological changes and vascular disturbances at doses that barely affected enzyme levels, suggesting that the primary mechanism involves attenuation of pancreatic circulatory disturbance rather than direct enzyme-release modulation [17]. The 7-day mortality was reduced by p-CPA pretreatment from 75.4% (vehicle) to 39.9% (200 mg/kg) and 22.2% (400 mg/kg) [17].

In the DINP model [20], risperidone (0.1–3.2 mg/kg s.c. b.i.d.) dose-dependently attenuated the entire pathological cascade. Serum IL-6 decreased from 4545.7 ± 1932.6 pg/mL (CDE controls) to 75.4 ± 18.9 pg/mL at 3.2 mg/kg (%Δ = −98.3%). Plasma amylase decreased from 28,332.9 ± 4704.1 to 9873.5 ± 2844.0 U/L (%Δ = −65.2%). Plasma lipase decreased from 2658.8 ± 396.8 to 1019.5 ± 264.1 U/L (−61.6%). Platelet counts, which decreased in CDE controls (0.78 ± 0.06 × 10^6^/mm^3^ vs. 1.06 ± 0.04 in normal chow), were dose-dependently restored by risperidone (to 1.24 ± 0.08 at 3.2 mg/kg). Histological scores for edema, infiltration, and necrosis were all dose-dependently reduced [20]. Day-3 mortality was 28.0% (16/65) in controls vs. 0% (0/65) at 3.2 mg/kg risperidone [20].

#### 3.3.3. Synthesis of the Serotonergic Strand

Across all serotonergic studies, directionality was consistently in favour of attenuation of biochemical injury (serum enzymes), histological damage (edema, infiltration, necrosis), and in severe models, systemic inflammation and mortality. The strongest quantitative evidence comes from the explicit dose–response datasets [16,17,20], where graded reductions in multiple endpoints track drug dose. The receptor-selectivity analysis [19] and the concordance of pharmacological depletion (p-CPA) with receptor antagonism strengthen the internal coherence of a 5-HT_2_A-mediated injury-amplification mechanism. However, necrosis scoring was inconsistent across models and laboratories, and survival was either not a primary endpoint or subject to survivor-restricted analyses.

### 3.4. Severe Procedural Models: L-17 and Survival Outcomes

The L-17 studies [31,32] evaluated whether stress-modulating neuroimmune activity can shift hard outcomes in high-severity, procedure-linked pancreatitis paradigms.

In the duct ligation/proserin model [31], Day-1 mortality was 10% (AP alone) vs. 0% (AP + L-17), and Day-7 mortality was 50% vs. 30%. L-17 prevented progressive granulocytosis: blood granulocytes in AP controls increased from 1.85 ± 0.64 × 10^9^/L (Day 1) to 7.45 ± 1.67 (Day 7), whereas in L-17-treated animals the increase was modest (2.57 ± 0.62 to 3.22 ± 1.64). Pancreatic tissue granulocyte infiltration was lower in L-17-treated animals (8.6 ± 4.6 vs. 12.96 ± 3.91 cells/mm^2^ on Day 1; 7.75 ± 6.2 vs. 20.76 ± 3.15 on Day 7), while monocyte and lymphocyte counts were higher, consistent with a shift toward reparative infiltrate composition. Cytokine trajectories showed complex patterns: TNF-α was paradoxically higher in L-17 groups on Day 1 (131.6 ± 32.2 vs. 53.23 ± 48.0 pg/mL), while IL-1β (54.45 ± 4.1 vs. 545.5 ± 441.2 pg/mL), IL-6, and IL-10 were lower, arguing against a simplistic “cytokine suppression” interpretation [31].

In the post-manipulation pancreatitis model [32], Day-7 mortality was 70% (controls) vs. 30% (L-17). Morphometric analysis of pancreatic tissue confirmed the same pattern: lower granulocyte counts and higher monocyte/lymphocyte counts in L-17-treated animals at both timepoints. Histologically, L-17-treated animals showed aborted inflammatory progression with reparative fibroblast activity and preserved Langerhans islets, whereas controls developed confluent necrosis, purulent peritonitis, and systemic inflammatory changes across multiple organs [32].

These mortality-centered findings sit alongside, rather than directly within, the enzyme-centered serotonergic pattern. The cytokine data suggest a composite neuroimmune shift rather than simple anti-inflammatory suppression, and the methodological reporting was insufficient for strong internal validity judgments.

### 3.5. Olanzapine in a Ferroptosis-Oriented Framework

Liu et al. [21] used an unbiased drug screening approach to identify olanzapine as a novel ferroptosis inhibitor in pancreatic acinar cells. In pancreas-specific Gpx4 knockout mice with cerulein-induced AP, olanzapine (5 mg/kg p.o.) reduced all measured pancreatitis parameters compared to vehicle in knockout mice: histological scores for acinar cell death, leukocyte infiltration, and interstitial edema were markedly attenuated; serum amylase, LDH, HMGB1, and pancreatic MPO, MDA, and trypsin activity were reduced; and ferroptosis markers (Ptgs2, Acsl4 mRNA) were decreased. Importantly, selective 5-HT_2_ receptor antagonists (risperidone, SB204741, SB242084) did not inhibit cerulein/trypsin-induced ferroptotic cell death in vitro, and knockdown of Htr2a, Htr2b, or Htr2c did not affect ferroptosis, establishing that olanzapine’s protective effect operates through an off-target antioxidant mechanism rather than serotonergic receptor blockade. Biomarkers of apoptosis (cleaved caspase-3) and necroptosis (p-MLKL) were unaffected by Gpx4 status or olanzapine treatment [21].

This axis is mechanistically distinct from the serotonergic strand and is best treated as proof-of-principle that ferroptosis-linked pharmacology constitutes a separate lever for modulating experimental AP severity.

### 3.6. Risk of Bias and Synthesis-Level Implications

**Figure 2:**
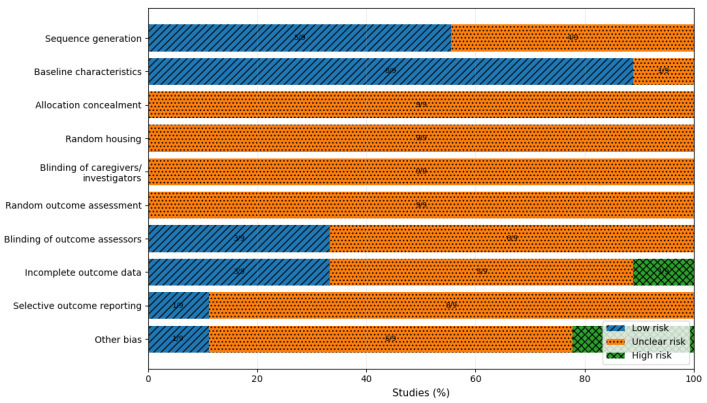
SYRCLE risk-of-bias summary across included studies (n = 9). Proportion of studies judged as low, unclear, or high risk for each SYRCLE domain.

Across the nine included reports, most SYRCLE domains were rated “Unclear,” driven by incomplete reporting of randomization, allocation concealment, housing procedures, and blinding. One report was judged at high risk for incomplete outcome data and other bias, primarily due to survivor-only quantitative endpoints and differential attrition. The risk-of-bias profile constrains inference: the evidence supports consistent directionality in several models and endpoints (particularly within the serotonergic strand), but it does not support high confidence in effect magnitude without improved reporting. The study characteristics (Table 1), condensed quantitative effect profile (Table 2), and risk-of-bias overview (Figure 2) are presented to make these constraints auditable.

## 4. Discussion

**Figure 3:**
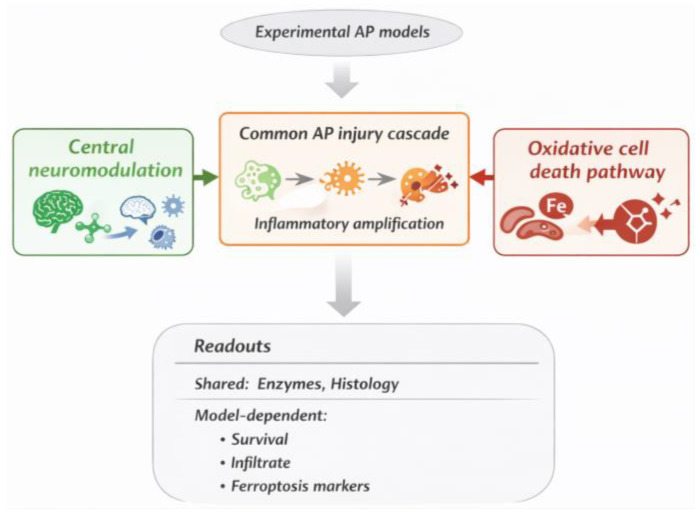
Central and cellular modulation of the injury cascade in experimental acute pancreatitis. Note: This schematic summarizes the organization of mechanistic pathways identified across the included studies. Experimental acute pancreatitis is represented as a common injury cascade (acinar injury → inflammatory amplification → pancreatic/systemic damage). Two non-equivalent classes of modulation are shown. On the left, central neuromodulation integrates serotonergic and stress-axis/neuroimmune mechanisms, which act on early injury amplification and are supported by the most coherent preclinical signal. On the right, cellular oxidative pathways (ferroptosis-linked mechanisms) represent a mechanistically distinct axis operating at the level of lipid peroxidation and cell death. Both routes converge on the same pathological cascade but produce partially overlapping and partially model-dependent readouts. Shared outcomes include pancreatic enzymes and histological injury, whereas survival, inflammatory-cell composition, and ferroptosis markers remain model-specific.

This systematic synthesis of nine controlled rodent in vivo studies indicates that neuromodulatory pathways can alter early injury trajectories in experimental AP. However, the evidentiary strength is not uniform across the three mechanistic strands: the serotonergic axis is supported by the most internally coherent and replicated pharmacological evidence, whereas the L-17 stress-axis strand and the ferroptosis-oriented olanzapine strand remain more exploratory and should not be interpreted as mechanistically equivalent (Figure 3).

Serotonergic axis. Across heterogeneous models and readout panels, the most reproducible effects were observed on short-term biochemical injury indices (serum amylase/lipase) and pancreatic histology scores, with directionality generally favouring attenuation under 5-HT_2_-preferring antagonists [16,17,18,19,20,30]. The receptor-selectivity analysis of Hamada et al. (2007) provides the most granular pharmacological evidence: selective 5-HT_2_A antagonists produced rank-order potencies paralleling their reported pKi values at the 5-HT_2_A receptor, whereas non-selective 5-HT_2_A/_2_B/_2_C antagonists were less potent than predicted by their 5-HT_2_A affinity alone, consistent with opposing vascular effects of 5-HT_2_B/_2_C blockade at the endothelial level [19]. Orthogonal evidence from pharmacological 5-HT depletion (p-CPA) converged with receptor-preferring antagonism on reduced hyperenzymemia, strengthening the internal coherence of the serotonergic “injury-amplification” axis without requiring overextension into definitive causal claims [17,19]. Early tissue-level evidence from Celiński et al. (1995), who documented significant reductions in pancreatic 5-HT and 5-HIAA during caerulein-induced AP in rats, provides independent biomarker-level plausibility for serotonergic involvement in the pancreatic injury milieu [15]. In contrast, clinically aligned endpoints—persistent organ failure, standardized extra-pancreatic injury, and adequately powered survival analyses—were sparsely reported, which limits any inference about clinically relevant effect magnitude [16,17,18,19,20,21,30,31,32,33]. This cautious interpretation also remains consistent with recent broader AP overviews emphasizing that acinar cell death programs, inflammatory amplification, and stage-specific therapeutic timing should be considered jointly rather than as isolated mechanistic layers [34].

The defensible mechanistic conclusion is therefore narrowly framed: serotonergic modulation robustly shifts early injury trajectories in rodent AP, while its influence on the downstream transition to sustained necrosis, systemic organ dysfunction, or durable survival benefit remains unresolved.

Stress-axis/neuroimmune axis. The L-17 evidence base is notable because it contributes a qualitatively different endpoint profile: two independent severe model settings reported sizeable absolute reductions in 7-day mortality under L-17, even though inflammatory panels showed complex and at times bidirectional cytokine patterns [31,32]. The morphometric data add specificity: L-17 shifted the composition of the pancreatic inflammatory infiltrate from granulocyte-dominant (pro-inflammatory, tissue-destructive) to monocyte/lymphocyte-dominant (reparative), which is mechanistically coherent with the observed survival benefit and histological evidence of accelerated granulation tissue formation [31,32]. However, the current corpus does not allow rigorous mechanistic attribution to specific serotonergic receptors versus broader stress-axis, autonomic, or neuroimmune pathways, and the methodological reporting was insufficient for strong internal validity judgments. This strand should therefore be interpreted as exploratory survival-oriented evidence rather than as target-resolving proof of mechanism.

Neuroimmune convergence. Beyond pharmacologic modulation, experimental work indicates that direct neuromodulatory interventions targeting the vagus nerve can also attenuate pancreatitis severity. Zhang et al. (2021) demonstrated that cervical vagus nerve stimulation reduced plasma amylase and lipase, decreased TNF-α, ameliorated histologic injury, and improved survival in a mouse duct-ligation AP model, with mechanistic evidence implicating α7 nAChR-positive macrophage populations [35]. These findings complement the present pharmacological synthesis by demonstrating that engaging the cholinergic anti-inflammatory pathway at the neural level can reproduce protective patterns paralleling those observed with serotonergic or stress-axis modulation, thereby strengthening the broader translational rationale for targeting neuroimmune circuits in AP [9,10,11,12,35]. Recent reviews of non-invasive vagus nerve stimulation further reinforce this translational perspective by integrating mechanistic evidence for cholinergic anti-inflammatory signaling with emerging clinical neuromodulation experience in inflammatory disorders [36]. Taken together, these data support the view that AP-relevant neuromodulation should be framed not only in receptor-pharmacology terms but also within a broader reflex-circuit model of inflammatory control [36].

Ferroptosis axis. The ferroptosis-oriented study adds a mechanistically distinct line: in a ferroptosis-sensitized framework, olanzapine was identified through unbiased drug screening as an antioxidant inhibitor of ferroptosis in pancreatic acinar cells, operating independently of serotonergic receptor blockade [21]. Crucially, selective 5-HT_2_ receptor antagonists and genetic knockdown of 5-HT_2_A/_2_B/_2_C receptors did not affect ferroptosis, confirming that olanzapine’s effect is off-target. However, olanzapine as a clinical drug carries well-recognized metabolic liabilities, and the study remains anchored to short-term pancreatic endpoints without addressing therapeutic windows, pharmacokinetic bridging, or clinically aligned systemic outcomes. At the tested murine dose, the finding is best interpreted as mechanistic proof-of-principle rather than dose-aligned repurposing evidence for clinical AP treatment [21]. This interpretation is strengthened by recent AP-focused literature that places ferroptosis within a broader network of regulated cell death programs relevant to disease severity and therapeutic targeting [37,38]. More recent mechanistic work has also identified the FAT10–NCOA4 axis as a regulator of ferroptosis in pancreatic acinar cells, further supporting the idea that ferroptosis in AP is an active pathogenic process rather than an isolated downstream epiphenomenon [39]. Accordingly, the present olanzapine signal is best interpreted as an entry point into an expanding ferroptosis-centered therapeutic literature rather than as a stand-alone drug-repurposing argument [37,38,39].

Pharmacovigilance paradox. A translational tension must be acknowledged. Pharmacoepidemiologic data on psychotropic drugs and AP risk are heterogeneous. Recent meta-analyses suggest that antidepressants and certain antipsychotic classes may be associated with modestly increased AP risk, although confounding by indication, metabolic comorbidity, and polypharmacy is difficult to fully exclude [22,23,24,25,26,27,28,40,41,42]. These signals argue against straightforward clinical repurposing of psychotropic drugs for AP without targeted safety-oriented translational work, and reinforce that the appropriate conclusion from the present preclinical evidence is target-level (5-HT_2_**A** signalling, stress-responsive pathways, ferroptosis-linked mechanisms) rather than drug-level.

Translational constraints. Two overarching limitations dominate inference. First, timing: all included interventions were administered prophylactically, peri-induction, or in the early post-induction window. No study evaluated delayed post-induction initiation, so the evidence addresses modulation of disease initiation/early escalation rather than treatment of established AP—a critical translational gap given that clinical AP presents after onset. Second, internal validity: SYRCLE domains were predominantly “unclear,” with attrition and survivor-restricted analyses introducing additional bias risk in at least one severe-outcome context. Publication bias assessment (Section 2.8) further suggests that reported effect magnitudes may be inflated by positive-result reporting. These constraints do not nullify the directionality signal but preclude robust effect-size inference.

Preclinical agenda. The next decisive step is not more prophylactic hyperstimulation work, but a design shift toward translational realism: post-induction dosing windows, severe models with standardized systemic outcomes (including organ injury and persistent dysfunction), adequate powering for survival, and transparent reporting. Future severe-model studies should also prespecify whole-cohort, intention-to-treat-style accounting of all allocated animals to avoid survivor-restricted inflation of apparent benefit. Serotonergic hypotheses should be tested with receptor-selective pharmacology and mechanistic controls distinguishing peripheral inflammatory modulation from central/autonomic effects. Stress-axis candidates require target-resolving experiments. Ferroptosis-oriented findings should be extended to systemic injury and therapeutic-window questions using safer, mechanism-specific ferroptosis inhibitors such as ferrostatin-1 or liproxstatin-1. Recent AP reviews centered on programmed cell death pathways support exactly this direction, namely, moving from descriptive ferroptosis association toward target-resolved intervention designs with clearer therapeutic timing and mechanistic specificity [37,38].

## 5. Limitations of the Evidence Base and Review Process

This review is constrained by the structure and reporting quality of the available preclinical literature. First, the evidence base is small (nine studies) and spans heterogeneous experimental platforms with variability in model class, timing, dosing, and outcome panels, precluding defensible quantitative pooling. Second, outcome extractability was incomplete: although figure digitization expanded numerical coverage, three studies relied predominantly on figure-based presentation of key endpoints, and digitized values cannot fully substitute for author-reported data when dispersion measures or group sizes are not recoverable. Third, the endpoint landscape was dominated by short-term pancreatic proxies, primarily serum enzymes and histology, whereas clinically aligned outcomes—persistent organ failure, standardized extra-pancreatic injury, and adequately powered survival—were inconsistently reported. Fourth, no included study evaluated delayed post-induction initiation, so the available evidence reflects modulation of early disease processes rather than treatment of established AP. Fifth, internal validity remains uncertain, as SYRCLE domains were predominantly rated as unclear, with attrition and survivor-restricted analyses contributing additional bias risk.

At the review-process level, Embase was not included among the searched databases, which may affect the completeness of pharmacological literature retrieval; however, a targeted Google Scholar coverage audit and systematic citation chasing from all included studies did not identify additional eligible intervention studies. The most recent included study dates to 2023, reflecting the apparent sparsity of published eligible intervention work in this niche during 2024–2025 rather than a search string deficiency; the targeted coverage audit and citation chasing did not recover any additional eligible records from this period.

We did not perform formal certainty-of-evidence grading, and the meta-analysis eligibility audit confirmed that no endpoint subset met the prespecified pooling criteria. Collectively, these limitations restrict inference about clinically relevant effect sizes, optimal timing, and mechanistic specificity.

Collectively, these limitations restrict inference about clinically relevant effect size, optimal treatment timing, and mechanistic specificity, and underscore the need for timing-explicit, endpoint-aligned, and transparently reported preclinical study designs.

## 6. Conclusions

This systematic review synthesizes nine controlled rodent studies (1992–2023) and shows that neuromodulatory pathways can influence the severity of experimentally induced AP. Three mechanistic signals emerge: a comparatively well-supported serotonergic 5-HT_2_/5-HT_2_A-oriented strand associated with reproducible reductions in biochemical and histological injury; an exploratory stress-axis/neuroimmune strand centered on L-17 with survival-oriented effects in severe models; and a mechanistically distinct ferroptosis-linked strand in which olanzapine acts through off-target antioxidant mechanisms.

Translational inference remains limited. The available evidence is dominated by prophylactic or peri-induction dosing, surrogate pancreatic endpoints, and incompletely reported methodology. Accordingly, the present corpus supports mechanism-level target prioritization and hypothesis generation, but does not support direct clinical repurposing claims.

Future work should prioritize delayed post-induction designs, clinically aligned systemic outcomes, and improved internal validity and reporting transparency.

## Figures and Tables

**Table 1:** Characteristics of included studies (n = 9).

Species/Strain/Sex	AP Model	Intervention	Timing Category	Severity	Study
Rat, Wistar, male	Cerulein hyperstimulation (20 µg/kg s.c. × 3, 1 h interval)	Ketanserin, ritanserin; 5-HT_2_ antagonism	Pre-induction	Mild-edematous	Oguchi 1992 [16]
Mouse, ICR, female	CDE diet (48 h); follow-up to day 7	Ketanserin, cyproheptadine, pindolol, NAN-190, ICS-205,930, M-840; p-CPA; serotonergic antagonism (5-HT_2_-focused)	Pre-induction (p-CPA)/Peri-induction (antagonists)	Severe-necrotizing	Yoshino 1997 [17]
Rat (Wistar, Wistar Bonn/Kobori), Mouse (ICR)	Cerulein; duct ligation; CDE diet; spontaneous chronic (WBN/Kob)	R-102444, R-96544; selective 5-HT_2_A antagonists	Pre-induction (cerulein, ligation)/Peri-induction (CDE)	Mild-to-severe (model-dependent)	Ogawa 2005 [18]
Mouse, ICR, female	Cerulein hyperstimulation (50 µg/kg i.p. × 5)	Risperidone, spiperone, ketanserin, AMI-193, MDL 11,939, metergoline, ritanserin, methysergide, SB204741, SB242084; p-CPA; DOI (agonist)	Pre-induction	Mild-edematous	Hamada 2007 [19]
Mouse, ICR, female	CDE diet (DINP); severe necrotizing AP	Risperidone; 5-HT_2_A-dominant antipsychotic	Peri-induction	Severe-necrotizing	Yamaguchi 2009 [20]
Mouse, C57BL/6, male (Gpx4 WT/KO)	Cerulein (50 µg/kg i.p. × 7) on ferroptosis-sensitized background	Olanzapine; identified as ferroptosis inhibitor via drug screening	Early post-induction	Moderate (cerulein) on sensitized background	Liu 2022 [21]
Mouse, NMR, male	Cerulein hyperstimulation (50 µg/kg i.p. × 5)	Tropisetron; 5-HT_3_ antagonist/α7 nAChR partial agonist	Peri-induction	Mild-edematous	Rahimian 2017 [30]
Rat, Wistar, male	Duct ligation + proserin; severe necrotizing AP	L-17 (thiadiazine); stress-modulating, putative SERT-oriented	Early post-induction	Severe-necrotizing	Sarapultsev 2018 [31]
Rat, Wistar, male	Post-manipulation pancreatitis (OPP model)	L-17 (thiadiazine); stress-modulating	Early post-induction	Severe-necrotizing	Rantsev 2023 [32]

Timing categories: pre-induction = completed before AP induction; peri-induction = overlapping with induction; early post-induction = first dose within hours of induction. No study evaluated delayed post-induction (≥24 h) therapeutic dosing. Detailed dosing schedules are reported in the text.

**Table 2:** Condensed quantitative effect profile of included neuromodulatory interventions in experimental acute pancreatitis. Percent change is shown relative to the matched disease-control group within the same study/model/timepoint stratum.

Study	Model	Intervention/Axis	Timing	Representative Quantitative Signals	Interpretive Note
Oguchi et al., 1992 [16]	Cerulein hyperstimulation (rat)	Ketanserin/ritanserin 5-HT_2_ antagonism	Pre-induction	Serum amylase −37.2% to −51.7% (ketanserin); −42.2% (ritanserin) Pancreas wet weight −10.8%	Clear dose–response reduction in hyperenzymemia in a mild-edematous model.
Yoshino and Yamaguchi, 1997 [17]	CDE diet severe necrotizing AP (mouse)	Ketanserin/cyproheptadine/p-CPA serotonergic modulation	Pre-/peri-induction	Best ketanserin arm: amylase −53.7%, lipase −59.3% p-CPA 7-day mortality −47.1% to −70.6%	Strong severe-model signal, but enzyme comparisons are survivor-restricted at Day 3.
Ogawa et al., 2005 [18]	Cerulein, ligation, CDE, and WBN/Kob models	R-102444/R-96544 selective 5-HT_2_A antagonism	Pre-/peri-induction	Cerulein arm: amylase −53.7%, lipase −57.7% (R-102444) CDE arm: amylase −85.5% (R-96544)	Cross-model support for a 5-HT_2_A signal, although designs and outcome contexts differ.
Hamada et al., 2007 [19]	Cerulein hyperstimulation (mouse)	Multiple antagonists + p-CPA receptor-selectivity analysis	Pre-induction	p-CPA: amylase −22.3%, lipase −29.3% Risperidone potency index: 42.5% (amylase), 60.5% (lipase) Inflammatory-cell infiltration −33.3% at 3.2 mg/kg	Most informative for rank-order receptor selectivity rather than a single pooled effect size.
Yamaguchi et al., 2009 [20]	CDE severe necrotizing AP (mouse)	Risperidone 5-HT_2_A-dominant antipsychotic	Peri-induction	IL-6 −98.7% Amylase −65.5%; lipase −75.9% Necrosis −44.4%; Day-3 mortality −100.0%	Strongest severe-model multi-endpoint signal in the corpus.
Rahimian et al., 2017 [30]	Cerulein hyperstimulation (mouse)	Tropisetron 5-HT_3_ antagonism/α7 nAChR-related	Peri-induction	Amylase −38.9%; lipase −50.0% MPO −38.6%; TNF-α −34.6%; IL-1β −41.7% Histology −45.0%	Broad attenuation across enzyme, cytokine, and histologic readouts.
Sarapultsev et al., 2018 [31]	Duct ligation + proserin severe AP (rat)	L-17 stress-axis/neuroimmune	Early post-induction	Day-7 mortality −40.0% Blood granulocytes −56.8% (Day 7) IL-1β −90.0%; IL-6 −51.3%	Survival-centered signal with nonuniform cytokine behavior (TNF-α increased on Day 1).
Rantsev et al., 2023 [32]	Post-manipulation pancreatitis (rat)	L-17 stress-axis/neuroimmune	Early post-induction	7-day mortality −57.1% Pancreatic granulocytes −62.7% Monocytes +81.3%; lymphocytes +183.6%	Supports inflammatory-cell redistribution rather than simple global suppression.
Liu et al., 2022 [21]	Cerulein AP in ferroptosis-sensitized background	Olanzapine ferroptosis-linked off-target	Early post-induction	Gpx4-KO arm: amylase −42.9%, MPO −50.0%, HMGB1 −50.0% Acinar cell death −50.0%; Ptgs2/Acsl4 −50.0%	Mechanistically distinct proof-of-principle; WT effects are weaker than in the sensitized arm.

## Data Availability

No new data were created or analyzed in this study. All data analyzed in this study are included in the published articles. Extracted datasets and analysis files are available from the corresponding author upon reasonable request.

## Supplementary Materials

The following supporting information can be downloaded at https://www.mdpi.com/article/10.3390/diseases14040145/s1. Table S1: Full endpoint-level extraction with disease-control means and percent change versus disease control.

## Abbreviations

AP, acute pancreatitis; CDE, choline-deficient ethionine-supplemented diet; DINP, diet-induced necrotizing pancreatitis; 5-HT, 5-hydroxytryptamine; 5-HIAA, 5-hydroxyindoleacetic acid; p-CPA, para-chlorophenylalanine; nAChR, nicotinic acetylcholine receptor; MPO, myeloperoxidase; HMGB1, high-mobility group box 1; LDH, lactate dehydrogenase; MDA, malondialdehyde; ITT, intention-to-treat; OSF, Open Science Framework; PRISMA, Preferred Reporting Items for Systematic Reviews and Meta-Analyses; SYRCLE, Systematic Review Centre for Laboratory Animal Experimentation; WT, wild type; KO, knockout.
