# Supplementary material for: Neuromodulatory Interventions in Experimental Acute Pancreatitis: A Systematic Review of Rodent Studies
Source: Diseases. 2026 Apr 16;14(4):145. doi: 10.3390/diseases14040145 (PMC13115382; doi:10.3390/diseases14040145)
Supplement: Supplementary file 1 [file diseases-14-00145-s001.zip › diseases-4245059-supplementary.pdf]

Supplementary Table S1. Full endpoint-level extraction with disease-control means and percent change versus disease control

Repeated values in the first four columns are merged vertically to reduce redundancy without loss of meaning. Blank cells indicate that a matched disease-control comparator was not computable within the same study/model/timepoint stratum.

Study	Model / stratum	Intervention	Timing	Endpoint	Timepoint	Disease control mean	Intervention mean	Unit	% Change
Oguchi et al. 1992	Cerulein hyperstimulation	Ketanserin	Pre-induction	Histology (edema + vacuolization)	5 h			Qualitative	
				Pancreas wet weight	5 h	0.74	0.66	g/100g BW	-10.8%
				Serum amylase		23043.3	14477	SU/dl	-37.2%
							12076.2		-47.6%
							11129		-51.7%
							11546.3		-49.9%
		Ritanserin	Pre-induction				13318.9		-42.2%
Yoshino and Yamaguchi 1997	CDE severe necrotizing AP	Cyproheptadine	Peri-induction		Day 3	46194	28805	IU/l	-37.6%
							36530		-20.9%
							20516		-55.6%
		Ketanserin				40725	43750		7.4%
							47227		16.0%
							18844		-53.7%
				Serum lipase		6576	7036		7.0%
							6187		-5.9%
							2677		-59.3%
		p-CPA	Pre-induction	7-day mortality	Day 7	75.4	39.9	%	-47.1%
						75.4	22.2	%	-70.6%
Ogawa et al. 2005	Mixed models (cerulein / ligation / CDE / WBN-Kobori)	R-102444	Peri-induction	Pancreatic amylase content	9 months age	3.3	6.9	x1000 IU/pancreas	109.1%
				Pancreatic amylase content		3.3	12.5	x1000 IU/pancreas	278.8%
				Pancreatic protein content		127	175.3	mg/pancreas	38.0%
						127	194.2		52.9%
				Pancreatic weight		193	215.3	mg/100g BW	11.6%
						193	224.9		16.5%

Study	Model / stratum	Intervention	Timing	Endpoint	Timepoint	Disease control mean	Intervention mean	Unit	% Change
			Pre-induction	Serum amylase	6 h	82	60	IU/ml	-26.8%
							48		-41.5%
							38		-53.7%
							85		3.7%
							75		-8.5%
							78		-4.9%
							52		-36.6%
				Serum lipase		5.2	3		-42.3%
						5.2	2.5		-51.9%
						5.2	2.2		-57.7%
		R-96544		Serum amylase	66 h	55	28		-49.1%
						55	12		-78.2%
						55	8		-85.5%
Hamada et al. 2007	Cerulein hyperstimulation	AMI-193		Potency index (amylase inhibition %)	1 h after 5th cerulein		4.4	% inhibition (potency index)	
		Ketanserin			1 h after 5th cerulein		29.1	% inhibition (potency index)	
		MDL 11,939			1 h after 5th cerulein		-6.6	% inhibition (potency index)	
		Risperidone	Pre-induction	Histology: Edema	5 h	1.95	1.75	Score (0-4)	-10.3%
						1.95	1.95		0.0%
						1.95	1.8		-7.7%
						1.95	1.9		-2.6%
				Histology: Inflammatory cell infiltration		2.1	2.05		-2.4%
						2.1	2.1		0.0%
						2.1	1.65		-21.4%
						2.1	1.4		-33.3%
				Histology: Necrosis		0.15	0.15		0.0%
						0.15	0		-100.0%
						0.15	0		-100.0%
						0.15	0		-100.0%

Study	Model / stratum	Intervention	Timing	Endpoint	Timepoint	Disease control mean	Intervention mean	Unit	% Change
				Potency index (amylase inhibition %)	1 h after 5th cerulein		42.5	% inhibition (potency index)	
		Spiperone		Potency index (amylase inhibition %)	1 h after 5th cerulein		34.8	% inhibition (potency index)	
		p-CPA		Plasma amylase	1 h after 5th cerulein	41174	32498	IU/L	-21.1%
						41174	31978		-22.3%
				Plasma lipase		1171.3	848		-27.6%
						1171.3	828.4		-29.3%
Yamaguchi et al. 2009	CDE severe necrotizing AP	Risperidone	Peri-induction	Day 3 mortality	Day 3	28	3.1	%	-88.9%
						28	1.5	%	-94.6%
						28	4.6	%	-83.6%
						28	0	%	-100.0%
				Histology: Edema		1.75	2.47	Score (0-4)	41.1%
						1.75	2.13	Score (0-4)	21.7%
						1.75	2	Score (0-4)	14.3%
						1.75	1.07	Score (0-4)	-38.9%
				Histology: Inflammatory cell infiltration		1.13	1.73	Score (0-4)	53.1%
						1.13	1.43	Score (0-4)	26.5%
						1.13	1.3	Score (0-4)	15.0%
						1.13	0.6	Score (0-4)	-46.9%
				Histology: Necrosis		2.75	2.47	Score (0-4)	-10.2%
						2.75	2.13	Score (0-4)	-22.5%
						2.75	1.87	Score (0-4)	-32.0%
						2.75	1.53	Score (0-4)	-44.4%
				Plasma amylase		28617.1	25000	U/l	-12.6%
						28617.1	22000	U/l	-23.1%
						28617.1	17000	U/l	-40.6%
						28617.1	9873.5	U/l	-65.5%
				Plasma lipase		4230.7	2200	U/l	-48.0%
						4230.7	2000	U/l	-52.7%
						4230.7	1550.8	U/l	-63.3%
						4230.7	1019.5	U/l	-75.9%
				Serum IL-6		5670.1	3700	pg/ml	-34.7%
						5670.1	2700	pg/ml	-52.4%
						5670.1	2500	pg/ml	-55.9%
						5670.1	75.4	pg/ml	-98.7%
Rahimian et al. 2017	Cerulein hyperstimulati on	Tropisetron	Early post-induction	Pancreatic IL-1beta	6 h after last cerulein	60	35	ug/100g tissue	-41.7%
				Pancreatic MPO activity		700	430	mU/g tissue	-38.6%
				Pancreatic TNF-alpha		130	85	ug/100g tissue	-34.6%
				Serum ALT		70	38	U/L	-45.7%
				Serum AST		220	130	U/L	-40.9%
				Serum amylase		18000	11000	U/L	-38.9%

Study	Model / stratum	Intervention	Timing	Endpoint	Timepoint	Disease control mean	Intervention mean	Unit	% Change
				Serum lipase		700	350	U/L	-50.0%
				Total histological score		4	2.2	Score (1-4)	-45.0%
		tropisetron	Early post-induction	Histological score	~10 h after first injection (6 h after last)	4	2	score (1–4)	-50.0%
Sarapultsev et al. 2018	Duct ligation + proserin severe AP	L-17	Early post-induction	Blood granulocytes	Day 1	1.85	2.57	10^9/l	38.9%
					Day 7	7.45	3.22		-56.8%
				Blood leukocytes	1 d	2.3	3.65		58.7%
					7 d	9.75	4.63		-52.5%
				Blood lymphocytes	1 d	0.45	0.92		104.4%
					7 d	1.86	1.16		-37.6%
				Blood monocytes	1 d	0.09	0.13		44.4%
					7 d	0.44	0.13		-70.5%
				Blood thrombocytes	Day 1	689.2	819.2		18.9%
					Day 7	1067.5	1097.1		2.8%
				Mortality	Day 1	10	0	%	-100.0%
					Day 7	50	30		-40.0%
				Plasma IL-10	Day 1	746.3	61.05	pg/ml	-91.8%
				Plasma IL-1beta	Day 1	545.5	54.45		-90.0%
				Plasma IL-6	Day 1	120	58.48		-51.3%
				Plasma TNF-alpha	Day 1	53.23	131.6		147.2%
Rantsev et al. 2023	Post-manipulation pancreatitis	L-17	Early post-induction	7-day mortality	Day 7	70	30	%	-57.1%
				Pancreatic granulocytes	Day 1	12.96	8.6	cells/mm2	-33.6%
					Day 7	20.76	7.75		-62.7%
				Pancreatic lymphocytes	Day 1	2.02	9.1		350.5%
					Day 7	2.68	7.6		183.6%
				Pancreatic monocytes	Day 1	1.7	3.2		88.2%
					Day 7	1.82	3.3		81.3%
				Pancreatic plasmocytes	Day 1	1	0.6		-40.0%
					Day 7	0.66	0.4	cells/mm2	-39.4%
Liu et al. 2022	Cerulein AP (WT and Gpx4-KO); Gpx4-KO arm	Olanzapine	Early post-induction	Histology: Acinar cell death (0-5)	12 h after last cerulein	2	2	Score (0-5)	0.0%
				Histology: Interstitial edema (0-5)		2	2.5	Score (0-5)	25.0%
				Histology: Leukocyte infiltration (0-5)		2.5	2.5	Score (0-5)	0.0%
				Pancreatic MDA		4	4	nM/g protein	0.0%
				Pancreatic MPO		1200	1000	U/mg	-16.7%
				Serum LDH		400	300	U/L	-25.0%
				Serum amylase		3500	3000	U/L	-14.3%
			Early post-induction	Acsl4 mRNA (AU)		4	4	AU	0.0%
				C-CASP3 (AU)		3.2	3.1	AU	-3.1%
				HMGB1		20	18	ng/mL	-10.0%

Study	Model / stratum	Intervention	Timing	Endpoint	Timepoint	Disease control mean	Intervention mean	Unit	% Change
				Hspa5 mRNA (AU)		2.5	2.5	AU	0.0%
				Ptgs2 mRNA (AU)		3	3	AU	0.0%
				Trypsin activity (fmol/mL)		200	90	fmol/mL	-55.0%
				Trypsin activity (fmol/mg)		1400	1500	fmol/mg	7.1%
				p-MLKL (AU)		2	2.1	AU	5.0%
	Cerulein AP (WT and Gpx4-KO); WT arm		Early post-induction	Histology: Acinar cell death (0-5)		2	1	Score (0-5)	-50.0%
				Histology: Interstitial edema (0-5)		2	1	Score (0-5)	-50.0%
				Histology: Leukocyte infiltration (0-5)		2.5	1.5	Score (0-5)	-40.0%
				Pancreatic MDA		4	2.5	nM/g protein	-37.5%
				Pancreatic MPO		1200	600	U/mg	-50.0%
				Serum LDH		400	250	U/L	-37.5%
				Serum amylase		3500	2000	U/L	-42.9%
			Early post-induction	Acsl4 mRNA (AU)		4	2	AU	-50.0%
				C-CASP3 (AU)		3.2	2	AU	-37.5%
				HMGB1		20	10	ng/mL	-50.0%
				Hspa5 mRNA (AU)		2.5	1.5	AU	-40.0%
				Ptgs2 mRNA (AU)		3	1.5	AU	-50.0%
				Trypsin activity (fmol/mL)		200	60	fmol/mL	-70.0%
				Trypsin activity (fmol/mg)		1400	900	fmol/mg	-35.7%
				p-MLKL (AU)		2	1.8	AU	-10.0%

Abbreviations: AP = acute pancreatitis; CDE = choline-deficient ethionine-supplemented; BW = body weight; MPO = myeloperoxidase; MDA = malondialdehyde; LDH = lactate dehydrogenase; IL = interleukin; TNF = tumor necrosis factor.

References.

1. Oguchi, H.; Terashima, M.; Koiwai, T.; Kawa, S.; Furuta, S.; Kobayashi, M.; Homma, T. Effects of the S2-serotonergic receptor antagonist, ketanserin, on cerulein-induced pancreatitis in the rat. Life Sci. 1992, 50, 733–737. https://doi.org/10.1016/0024-3205(92)90476-6.
2. Yoshino, T.; Yamaguchi, I. Possible involvement of 5-HT2 receptor activation in aggravation of diet-induced acute pancreatitis in mice. J. Pharmacol. Exp. Ther. 1997, 283, 1495–1502.
3. Ogawa, T.; Sugidachi, A.; Tanaka, N.; Fujimoto, K.; Fukushige, J.; Tani, Y.; Asai, F. Effects of R-102444 and its active metabolite R-96544, selective 5-HT2A receptor antagonists, on experimental acute and chronic pancreatitis: Additional evidence for possible involvement of 5-HT2A receptors in the development of experimental pancreatitis. Eur. J. Pharmacol. 2005, 521, 156–163. https://doi.org/10.1016/j.ejphar.2005.08.033.
4. Hamada, K.; Yoshida, M.; Isayama, H.; Yagi, Y.; Kanazashi, S.; Kashihara, Y.; Takeuchi, K.; Yamaguchi, I. Possible involvement of endogenous 5-HT in aggravation of cerulein-induced acute pancreatitis in mice. J. Pharmacol. Sci. 2007, 105, 240–250. https://doi.org/10.1254/jphs.FP0071049.

5. Yamaguchi, I.; Hamada, K.; Yoshida, M.; Isayama, H.; Kanazashi, S.; Takeuchi, K. Risperidone attenuates local and systemic inflammatory responses to ameliorate diet-induced severe necrotic pancreatitis in mice: It may provide a new therapy for acute pancreatitis. J. Pharmacol. Exp. Ther. 2009, 328, 256–262. https://doi.org/10.1124/jpet.108.141895.
6. Rahimian, R.; Moini Zanjani, T.; Mehr, S.E.; Pourgholami, M.H.; Hosseinzadeh, H. Tropisetron, a 5-HT3 receptor antagonist, attenuates cerulein-induced acute pancreatitis in mice. Eur. J. Pharmacol. 2017, 809, 84–92. https://doi.org/10.1016/j.ejphar.2017.05.032.
7. Sarapultsev, A.; Chupakhin, O.; Rantsev, M.; Sarapultsev, P.; Danilova, I.; Medvedeva, S.; Sidorova, L.; Tseitler, T.; Brilliant, S.; Tseilikman, V. Effects of 1,3,4-thiadiazine compound with antidepressant properties in ligation model of acute pancreatitis. Gen. Physiol. Biophys. 2018, 37, 549–562. https://doi.org/10.4149/gpb_2018012.
8. Rantsev, M.; Sarapultsev, A.; Medvedeva, S.; Danilova, I.; Sarapultsev, P. A thiadiazine compound reduces mortality in a post-manipulation pancreatitis model in rats. J. Surg. Res. 2023, 283, 120–129. https://doi.org/10.1016/j.jss.2022.11.032.
9. Liu, K.; Liu, J.; Zou, B.; Li, C.; Zeh, H.J.; Kang, R.; Kroemer, G.; Huang, J.; Tang, D. Trypsin-mediated sensitization to ferroptosis increases the severity of pancreatitis in mice. Cell. Mol. Gastroenterol. Hepatol. 2022, 13, 483–500. https://doi.org/10.1016/j.jcmgh.2021.09.008.
